# Impulse-Control Disorders in Parkinson’s Disease: A Meta-Analysis and Review of Case–Control Studies

**DOI:** 10.3389/fneur.2018.00330

**Published:** 2018-05-22

**Authors:** Helge Molde, Yasaman Moussavi, Stine Therese Kopperud, Aleksander Hagen Erga, Anita Lill Hansen, Ståle Pallesen

**Affiliations:** ^1^Department of Clinical Psychology, University of Bergen, Bergen, Norway; ^2^Norwegian Centre for Movement Disorders, Stavanger University Hospital, Stavanger, Norway; ^3^Department of Psychosocial Science, University of Bergen, Bergen, Norway

**Keywords:** Impulse-control disorders, Parkinson’s disease, case–control, meta-analysis, dopamine agonists

## Abstract

**Background:**

Although several case–control studies on the prevalence of Impulse-Control Disorders (ICDs) in Parkinson’s Disease (PD) have been conducted, no meta-analytic study on this topic has previously been published. Thus, knowledge about the overall prevalence rate of ICD in PD and factors that might moderate this relationship is lacking.

**Method:**

Prevalence studies of ICDs in PD were identified by computer searches in the MEDLINE, PsycINFO, and Web of Science databases, covering the period from January 2000 to February 2017. Data for *N* = 4,539, consisting of 2,371 PD patients and 2,168 healthy controls, representing 14 case–control studies were included. Estimation of the odds ratio (*OR*) of ICDs in PD compared to healthy controls was conducted using random-effects models. Mixed-effects models were applied in the moderator analysis of heterogeneity. Publication bias was estimated using a contour-enhanced funnel plot, the Rüker’s test, and fail-safe *N* test for estimating the number of potential missing studies.

**Results:**

Overall, the results showed significantly higher ratios for several ICDs in PD compared to healthy controls with the estimated overall *OR*s ranging between 2.07, 95% CI [1.26, 3.48], for having any ICDs, and 4.26, 95% CI [2.17, 8.36], for hypersexuality. However, the random-effects results for shopping were non-significant, though the fixed-effects model was significant (*OR* = 1.66, 95%CI [1.21, 2.27]). The testing of potential moderator variables of heterogeneity identified the following two variables that were both associated with increased risk: being medically treated for PD and disease duration. The results must be interpreted with some caution due to possible small-studies effect or publication bias.

**Conclusion:**

Individuals with PD seem to have a significantly greater risk of suffering from ICDs compared to healthy controls. Gambling, hypersexuality, eating, punding, and hobbying are all ICDs significantly associated with PDs being medically treated for PD.

## Introduction

Impulse control disorders (ICDs) are a collective term for non-motor symptoms that include pathological gambling, compulsive shopping, hypersexuality, and binge eating (1). In addition, behavioral disorders such as hobbyism (including pathological internet use), punding, and walkabout have been reported in patients with Parkinson’s disease (PD) (2–4). ICDs are behavioral addictions marked by an uncontrollable and irresistible drive or temptation to perform an action, even though this may be adverse to oneself or others. Such behaviors are often performed without the patient experiencing distress (5). ICDs are more frequently reported in PD patients compared to healthy control subjects, or the general population (6). ICDs in PD were first presented in a case report from 2000 (7).

Impulse control disorders have different levels of severity. Pathology is defined by its interference with financial, personal, family, and/or professional life. The addictive behavior is often time-consuming, and can cause significant distress and impinge on the quality of life (6, 8). ICDs are also associated with depression and low activity level (9, 10). Additionally, PD patients with ICDs experience more motor complications, although this may be due to medication (11).

Although ICDs are considered a common non-motor complication of PD, frequency estimates range from approximately 14–60% in PD (12, 13). Early investigations into ICDs in PD typically used screening tools and diagnostic criteria validated for the four most common ICDs (gambling, eating, hypersexuality, and shopping) in PD (13). With the introduction of the PD Impulsive-Compulsive Disorders Questionnaire (QUIP) (14), evaluation of the full range of impulsive and compulsive behaviors became more common and led to an increase in frequency estimates in later studies (15, 16). However, the estimated frequency of ICDs in PD still varies between cohorts, possibly due to differences in recruitment strategies between studies (15, 17, 18). Finally, the use of self-assessment of ICDs may serve as a possible bias in many studies, especially when estimating the frequency of hypersexuality, punding, and compulsive medication use (19).

Impulse control disorders in PD seem to be linked to certain risk factors: young age, male gender, being unmarried, higher education, novelty-seeking personality traits, personal or family history of addictions prior to PD diagnosis, and comorbid psychiatric disorders (11, 13, 18, 20–23). Hypersexuality and gambling seem to be more prevalent among males, while a female preponderance has been shown for compulsive shopping and binge eating (11, 13, 24). Type of ICD is further likely to be influenced by cultural or ethnic differences, genes, and access (e.g., to casinos) (11, 13, 25).

Dopaminergic medication, especially dopamine agonists (DAs) are associated with higher frequencies of ICDs (26–32). Although PD patients report ICDs more frequently than controls, this difference is not observed among unmedicated PD patients, arguing for a potential relation between ICDs and pharmacotherapy (33). Indeed, ICDs have consistently been associated with dopamine replacement therapy, such as DA. Hence, DA treatment seems to be a risk factor in the development of ICDs among Parkinson patients, although patients, family members, and physicians may disregard medication side effects and misinterpret them as changed behavior, or a psychiatric disorder (34).

Although several narrative reviews on ICDs related to PD have been published (1, 35, 36), a quantitative meta-analysis that summarizes the existing research could extend earlier reviews by providing overall prevalence estimates (precision estimates) as well as identifying significant moderator variables. Against this backdrop, we conducted a meta-analysis of ICDs in PD aiming to determine the overall prevalence of different ICDs in PD patients in comparison with healthy controls across case–control studies. The second aim was to model how different moderators, e.g., the severity of Parkinsonism (H–Y stage) (37) in a study, are related to ICD prevalence rates. The main research questions are: are ICDs significantly associated with PDs in case–control studies? If so, what moderates the level of association?

## Method

### Search Strategy, Inclusion and Exclusion Criteria

We conducted a systematic search and literature review following the PRISMA guidelines (38) in MEDLINE, Web of Science and PsycINFO for articles published between the year 2000 and January 19, 2017. The following keywords: Parkinson* AND “impulse control disorder*” OR impulsiv* OR gambl* OR shop* OR binge* OR “eating” OR “punding” OR “sex” OR hypersex* OR “hobbying” OR “buying” OR “gaming” OR “internet addiction*” OR “kleptomania” OR “skin picking” OR “trichotillomania” OR “intermittent explosive disorder” OR “pyromania” OR “walkabout” OR “medication” OR “dopamine dysregulation syndrome” OR “compulsive medication use” OR “repetitive behavior*” OR “stereotypical movement disorder*” OR “behavioral addiction” AND prevalen* OR inciden* OR frequen* were used for the search.

The studies were included if they fulfilled the following criteria: (a) the full article was published in English, (b) the article was published between the year 2000 and January 19, 2017, (c) the article had to contain original data on prevalence rates for ICDs and/or impulse-control disorders and related behaviors, and (d) the article had to be a case–control study or a case–control poster.

Together the search generated a total of 3,359 articles. References for 391 articles were further screened by their abstracts, as well as their method and results sections for inclusion eligibility.

**Figure 1:**
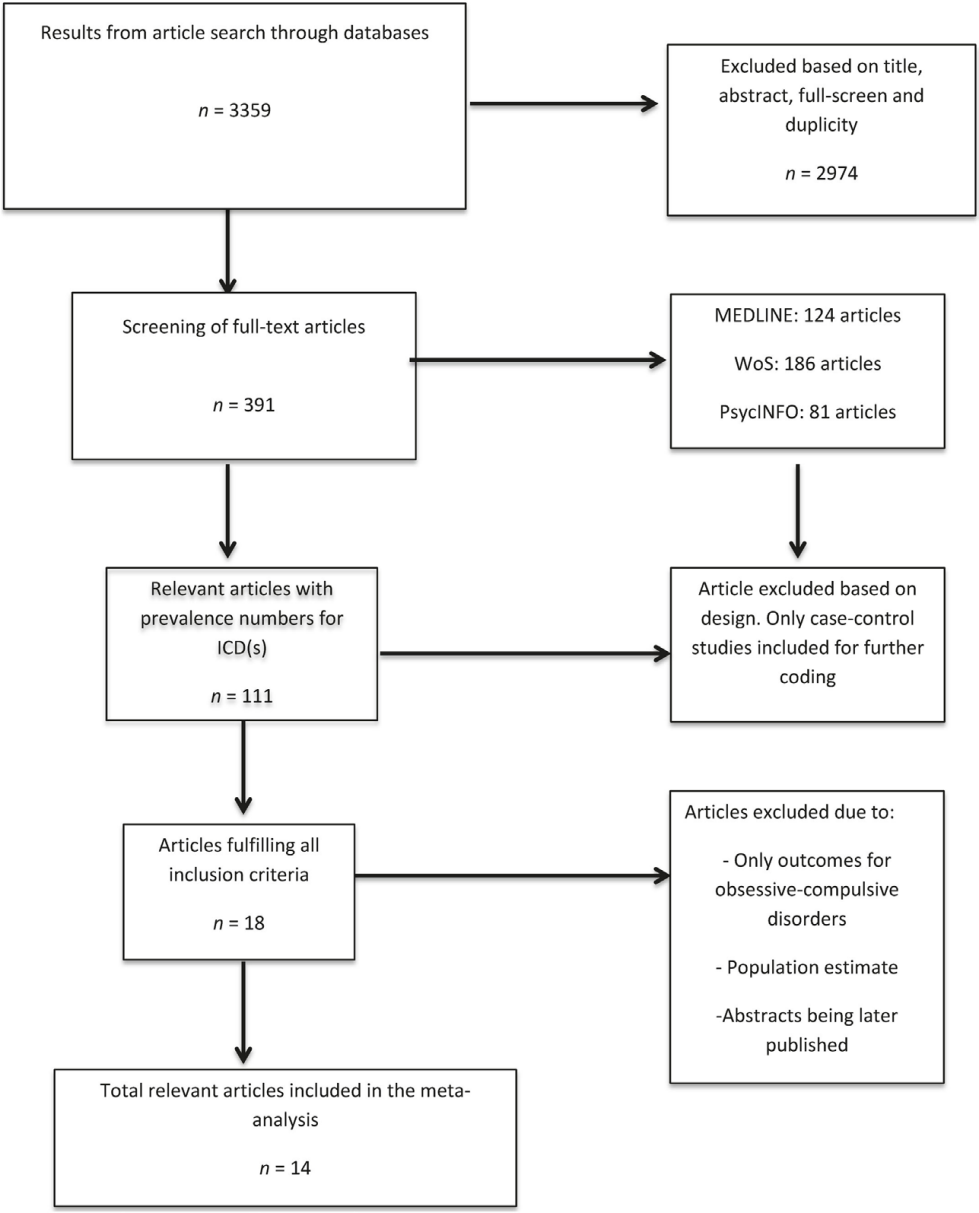
Flow diagram of literature search.

From this pool, 17 articles were retained for further evaluation of relevance. Of these, four articles were excluded due to: (1) measuring only outcomes for obsessive–compulsive disorders (39); (2) using a population estimate as a control condition (40); (3) a published poster (41) later published as an article (42); and (4) a published poster (43) later published as an article with an updated *N* (33). In addition, data from one article included was provided by one of the coauthors (Aleksander Hagen Erga) and published online first in February 2017 (15). Thus, a total of 14 case–control studies met the inclusion criteria (15, 16, 33, 42, 44–54). See Figure 1 for details.

The total participant population was *N* = 4,538, consisting of 2,371 PD patients and 2,168 healthy controls. For further details regarding the specific studies’ characteristics of response rate, the severity of Parkinsonism or H–Y stage, mean age, sample size, duration of illness, number of patients on DAs, as well as the proportion of Parkinson patients versus healthy controls, see Table 1.

**Table 1:** Characteristics of case–control studies on the prevalence of ICD(s) in Parkinson patients and healthy controls.

Reference	Country	Sample type	*N* (PP)	Response rate%	Hoehn–Yahr	Age mean	Sample size (male)	Duration of Parkinson	*n* DA	*n* Levodopa	ICD + PP%	*N* (HC)	ICD +HC%
Antonini et al. (44)	Italy	Patients/clinic	103	100	1.5	60.5	67	15.4	0	0	16.5	100	20
Avanzi et al. (45)	Italy	Patients/Clinic	98	100	2.26	69.9	49	7.6	13	44	6.12	392	0.25
de Chazeron et al. (46)	France	Patients/Clinic	115	72.3	–	66.9	60	7.4	65	101	14.4	115	17
Erga et al. (15)	Norway	NRR/MR	155	80.7	2.2	70.4	60	7.4	78	103	30.4	159	11.9
Gescheidt et al. (47)	Czech Republic	Patients/Clinic	49	100	2.29	47	69.4	11	38	40	26.5	38	10.5
Giladi et al. (48)	Israel	Patients/Clinic	203	95	2.7	67.5	63.2	9.6	115	157	14	190	0
Isaias et al. (49)	Italy	Patients/Clinic	50	100	–	65	62	9	50	48	28	100	20
Perez-Lloret et al. (42)	France	Patients/Clinic	203	100	8.95	67	62	9	161	178	25	52	0
Rodríguez-Violante et al. (16)	Mexico	Patients/Clinic	300	100	2.27	61.7	54.3	–	172	220	25.6	150	16.6
Sarathchandran et al. (50)	India	Patients/Clinic	305	100	2.5	58	71.1	7.6	149	244	31.6	234	15.4
Vela et al. (53)	Spain	Patients/Clinic	87	96.6	2	47	60.7	5	68	54	58.3	85	32.2
Valença et al. (52)	Brazil	Patients/Clinic	152	100	2.5	67.3	56.6	7.2	39	138	18.4	212	4.2
Weintraub et al. (33)	USA/Multinational	Patients/Clinic	168	90.3	2	61.5	71.4	^a^	0	0	18.5	143	20.3
Weintraub et al. (54)	USA/Multinational	Patients/Clinic	423	100	1.97	61.7	65	6.65	0	0	21	196	18

*Data that were not reported are presented with a dash. PP, Parkinson’s disease patients; DA, dopamine agonist; ICD+, Impulse-control disorder positive; HC, healthy control participants*.

*^a^Within 2 years*.

### Coding Procedures

A coding scheme was developed and used by two of the authors (Yasaman Moussavi and Stine Therese Kopperud) who coded the studies and were trained to ensure a common understanding of the coding scheme. Potential disagreements were resolved by discussing the topic with a third author (Helge Molde) in order to reach an agreement. The coding scheme comprised a number of descriptive codes such as study ID (numeric), coder (1 or 2), journal of publication, publication year, country and continent, ethical approval, and conflicts of interest (yes/no). Furthermore, the coding scheme consisted of information regarding the specific data and findings; total sample size for PD patients, sample size for healthy controls, measurement instrument for the ICD (self-report, interview, objective measure of a diagnosis, clinical testing, local medical registry, local administrative registry, national registry/database), mean Parkinson stage (Hoehn–Yahr), mean age, sex, duration of PD and whether patients with dementia were included or not. Finally, the coding scheme covered information about medical treatment of PD: *n* participants on levodopa as well as *n* participants on DAs. In addition, we also included the mean UPDRSIII motor score, being medically treated versus “*de novo*” PDs (treated = 1) and mean onset of PD (calculated as “age minus duration of PD”) as potentially moderators. The last section of the coding scheme included the prevalence of the total and individual ICDs. These were reported as numerals, as well as percentages. The last section was identical to that of the healthy control group.

The ICDs that were listed in the coding scheme were: Gambling, shopping, binge eating, punding, hypersexuality, hobbying, gaming, internet addiction, kleptomania, skin picking, trichotillomania, intermittent explosive disorder, pyromania, walkabout, compulsive medication use, repetitive behavior, stereotypical movement disorder, and dopamine dysregulation syndrome.

### Description of Studies

Two studies were multinational and included a number of European and US sites. Eight studies were from Europe, one was from South America, three from North America, one from the Middle East, and one from the southwest part of Asia (India). See Table 1 for details. Different ICDs were studied with different frequency. For gambling there were 14 relevant articles, eating 10 articles, hypersexuality 13 articles, shopping 12 articles, punding 8 articles, and finally hobbyism 6 articles.

### Statistical Analysis

We conducted a meta-analysis for each ICD separately, in addition to an analysis with an estimate of any/composite ICDs using random-effect models. For all models and outcomes, a first step in the analysis was to fit a random-effects model. See Table 2 for an overview of the results. This model assumes variance or heterogeneity between studies, in addition to within-study measurement error (55). This is a null-model without predictors. Tau2 is a measure of between-study variance, and a Tau2 = 0 would imply that there is no variance between the studies. A significant *Q*-statistics implies significant between-study variance, or that there are significant differences between the studies in the overall estimate of the mean effect. The *I*^2^ statistics [100% × (*Q − df*./*Q*)] is a measure of percentage of variability in effect sizes that is a result of true differences between the studies. Hence, *I*^2^ is an index of percentage of unexplained between-study variance of the mean estimate. A rough guide to interpret *I*^2^ is that percentages of around 25, 50, and 75% imply low, medium, and high heterogeneity, respectively. Also, the *I*^2^ index and the Tau2 are directly related, as the higher the between-study variance (Tau2), the higher the *I*^2^ index (56).

**Table 2:** Random-effects model.

	Total ICD	95% CI	Gambl.	95% CI	Hypers.^a^	95% CI	Shopping	95% CI	Eating	95% CI	Punding	95% CI	Hobbying	95% CI
Intercept (β0)^b^	2.10	[1.26, 3.48]	2.70	[1.56, 5.67]	4.26	[2.17, 8.36]	1.80	[0.99, 3.27]	2.32	[1.15, 4.68]	3.02	[2.31, 3.96]	1.72	[0.48, 6.18]
Heterogeneity														
Tau2	0.27		0.02		0.51		0.41		0.58		0.00		0.78	
*I*^2^ (%)	70.7	[47.1, 83.7]	1.70	[0.00, 55.8]	42.4	[0.00, 70.0]	50.8	[4.70, 74.6]	66.8	[35.2, 82.9]	0.00	[0.00, 21.3]	81.1	[59.5, 91.2]
H	1.85	[1.37, 2.48]	1.01	[1.00, 1.50]	1.32	[1.00, 1.83]	1.43	[1.02, 1.98]	1.73	[1.24, 2.42]	1.00	[1.00, 1.13]	2.30	[1.57, 3.37]

*CI, confidence interval; ICD, impulse-control disorder*.

*^a^Hypers. = hypersexuality*.

*^b^Odds ratio estimate*.

After assessing heterogeneity, potential moderators were regressed using mixed multivariate models. All moderators, except being medically treated for PD, were mean-centered in order to easily interpret the intercept (57). All results in the text and tables are reported as odds ratio (*OR*). The *OR* was calculated in such a way that an *OR* above 1 indicates higher odds for the Parkinson group having an ICD, in comparison to the odds for the healthy control group.

Some moderator variables had missing data. Data for these variables was imputed using “multivariate imputation by chained equations” through the mice package in R (58).

Small-study effects (or “publication bias”) were estimated using a *contour-enhanced funnel plot*. A funnel plot is a plot of each trial’s *OR* against the standard error. The plot should be shaped like a funnel if no publication bias is present (59). The contour-enhanced plot may help in differentiating between asymmetry due to publication bias and/or other reasons. Different gray areas correspond to different levels of significance, and studies missing in the white region are due to publication bias (e.g., no significant studies). Studies missing in the gray areas are missing due to other reasons (60). Furthermore, evaluating funnel plot asymmetry for binary data, Sterne et al. recommended the parametric Harbord’s test, the Peter’s tests and/or the Rüker’s test when Tau2 < 0.1, and only use of the Rüker’s test when Tau2 > 0.1 (61). Hence, we used the Rüker’s test, evaluating missing studies due to small-studies effects.

In addition, the Rosenthal’s fail-safe *N* test was applied, estimating how many non-significant studies are needed in order to have a non-significant overall result (62).

The residuals of the fitted models were inspected for normality using QQ-plots. Statistical analyses were conducted using the R packages *meta* (63) and *metafor* (64). Estimating the random-effects models, the Manzel–Haenszel estimator was used as estimator for the *OR* estimate, with Hartung-Knapp adjustment for small-studies effects. In addition, the DerSimonian–Laird estimator was used for estimating Tau2. All moderator analyses were conducted using a restricted maximum-likelihood estimator (REML), which is the default in the *metafor* package (64) in R (65).

## Results

Of all studies identified, 14 finally met all inclusion criteria and were included for further analysis. Eleven studies reported which DAs and mean dose were given to the patients, and nine studies reported levodopa usage and mean dose. Of all the patients having PD disease in this meta-analysis, 948 patients were on DA treatment, and 1,327 patients were on levodopa.

### Any ICDs

#### Random-Effects Model

**Figure 2:**
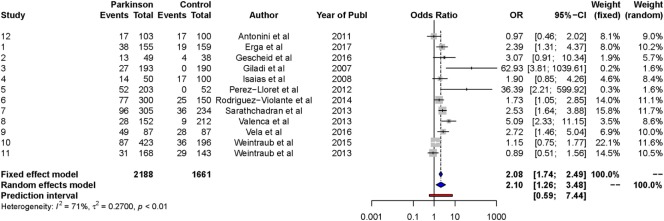
Forest plot of any ICDs.

The results from the random-effects model on *any ICDs* showed a significant *OR* estimate of point 2.10, 95% CI [1.26, 3.48]. The *Q*-statistics were significant (*Q* = 37.5, *p* < 0.0001), indicating significant heterogeneity between the studies. The between-study variance, Tau2, was 0.27, and the percentage of unexplained between-study variance *I*^2^ = 70.7, 95% CI [47.1, 83.7]. This indicated high unexplained between-study variance with respect to the total number of ICDs. See Figure 2 for a forest plot of the results.

**Figure 3:**
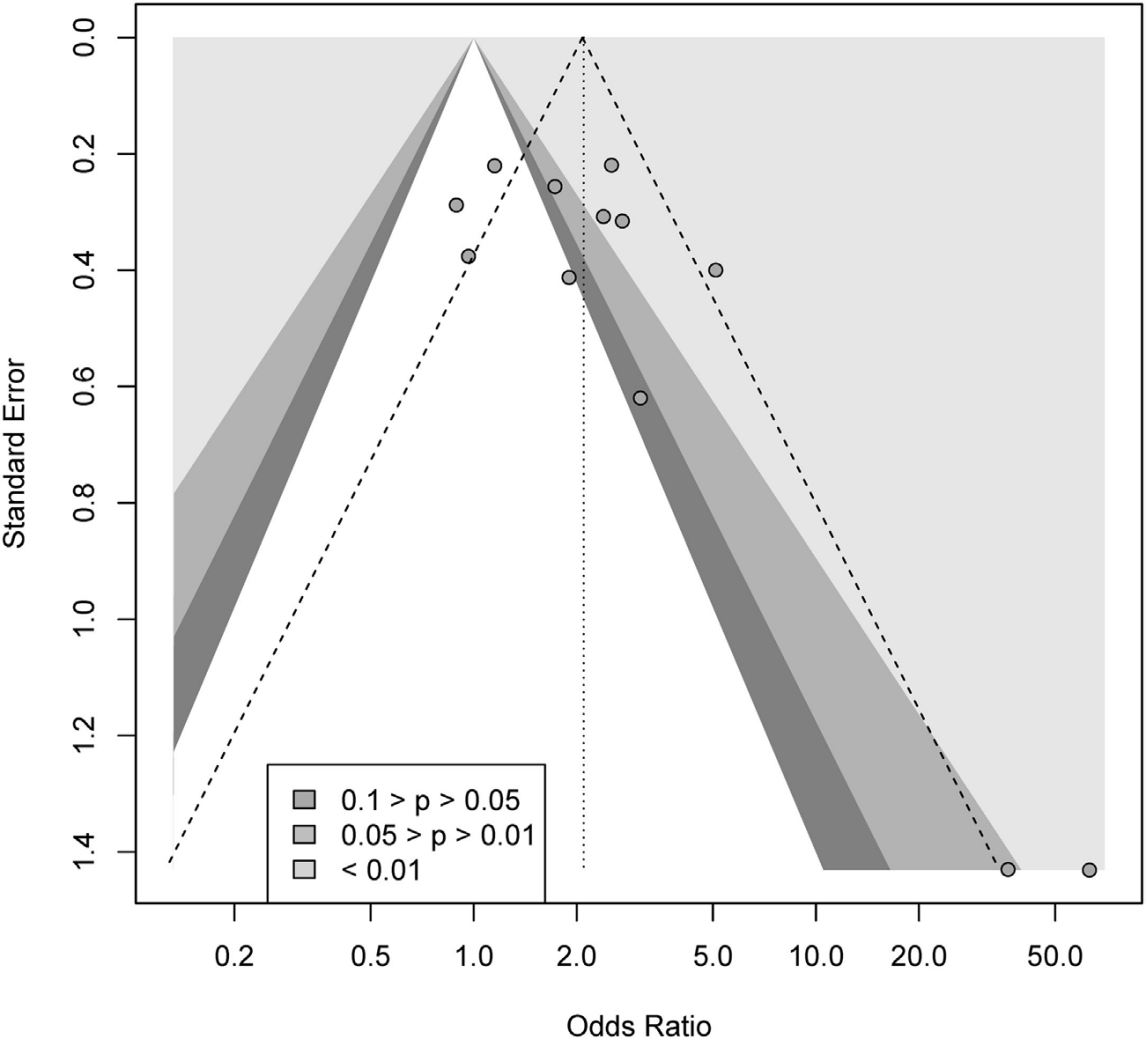
Contour-enhanced funnel plot for gambling.

We conducted a contour-enhanced funnel plot estimating publication bias. As seen from Figure 3, there seems to be a greater number of studies with low standard error lacking in the upper gray area, as compared to the number of studies (2) with large standard errors lacking in the lower white area, in order to create more balance in the figure. Thus, the former may indicate a small-study effect for other reasons than publication bias, while the latter indicate missing studies due to publication bias. As such, the Rüker’s test was non-significant (*t* = 0.69, *p* > 0.05), indicating no publication bias.

The Rosenthal’s fail-safe *N* test was significant (*p* < 0.0001), indicating that 226 non-significant studies would be needed in order for the random-effects model to be non-significant.

The univariate testing of possible moderators of heterogeneity resulted in two significant univariate models: treated PDs and disease duration. Hence, these parameters were included in a mixed multivariate model.

#### Mixed-Effects Model

The final model included only medically treated PDs as a moderator. The results from the meta-regression analysis showed a non-significant log-*OR* point estimate of β0 = 0.03 (*OR* = 1.03, 95% CI [0.67, 1.59]). The intercept β0 refers in this model to *de novo* patients, or non-medically treated patients. The test for residual heterogeneity indicated non-significance, QE = 15.0, *p* = 0.13. The overall moderator model was significant, QM = 13.90 (*p* = 0.004). Being medically treated for PD was significant at the 0.01 level, *OR* = 2.46, 95% CI [1.44, 4.22].

The model accounted for 100% (*R*^2^) of the heterogeneity, and the percentage of residual heterogeneity, *I*^2^, was 0%. Tau2, or residual heterogeneity, was 0.00 (*SE* = 0.04). The QQ-normal plot indicated a normal distribution of the residuals.

### Gambling

#### Random-Effects Model

**Figure 4:**
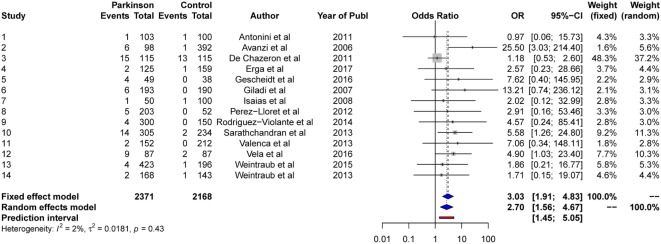
Forest plot of gambling.

The results from the random-effects model on *gambling* showed a significant *OR* estimate of point 2.70, 95% CI [1.56, 4.67]. The *Q*-statistics were non-significant (*Q* = 13.3, *p* > 0.05), indicating non-significant heterogeneity between the studies. The between-study variance, Tau2, was 0.02, and the percentage of unexplained between-study variance *I*^2^ = 1.70%, 95% CI = [0.00, 55.8]. This indicates almost no unexplained between-study variance with respect to gambling. See Figure 4 for a forest plot of the results.

**Figure 5:**
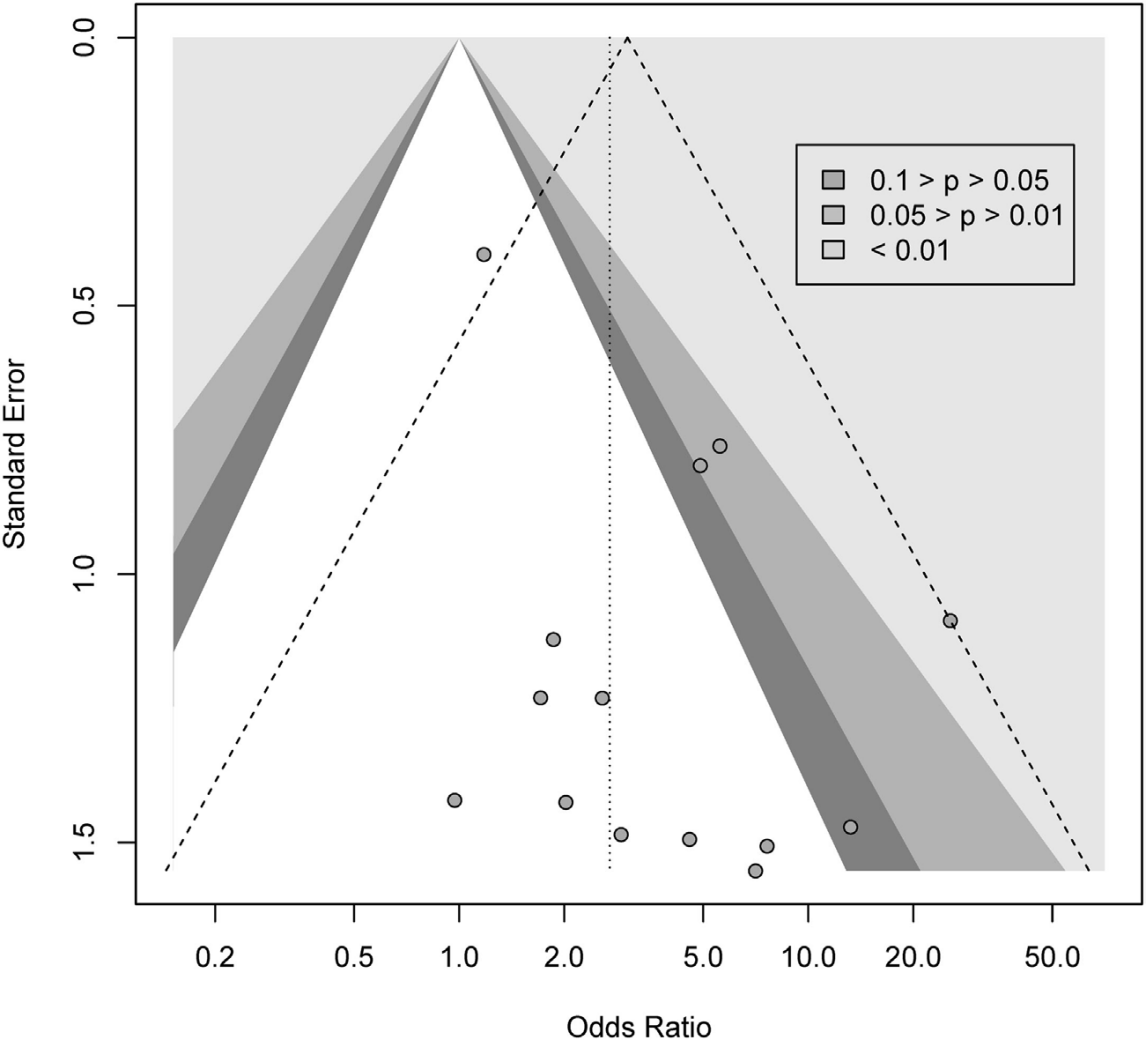
Contour-enhanced funnel plot for gambling.

We conducted a contour-enhanced funnel plot estimating publication bias. As seen from Figure 5, there seem to be lot more studies lacking in the lower white area, in comparison to the gray area, in order to create more balance in the figure. Thus, this may indicate a possible publication bias for gambling. However, the Rüker’s test was non-significant (*t* = 1.01, *p* > 0.05).

Rosenthal’s fail-safe *N* test was significant (*p* < 0.0001), indicating that 81 non-significant studies would be needed for the random-effects model to be non-significant.

As there was no significant between-study variance or heterogeneity, no further analyses were conducted for gambling.

### Hypersexuality

#### Random-Effects Model

**Figure 6:**
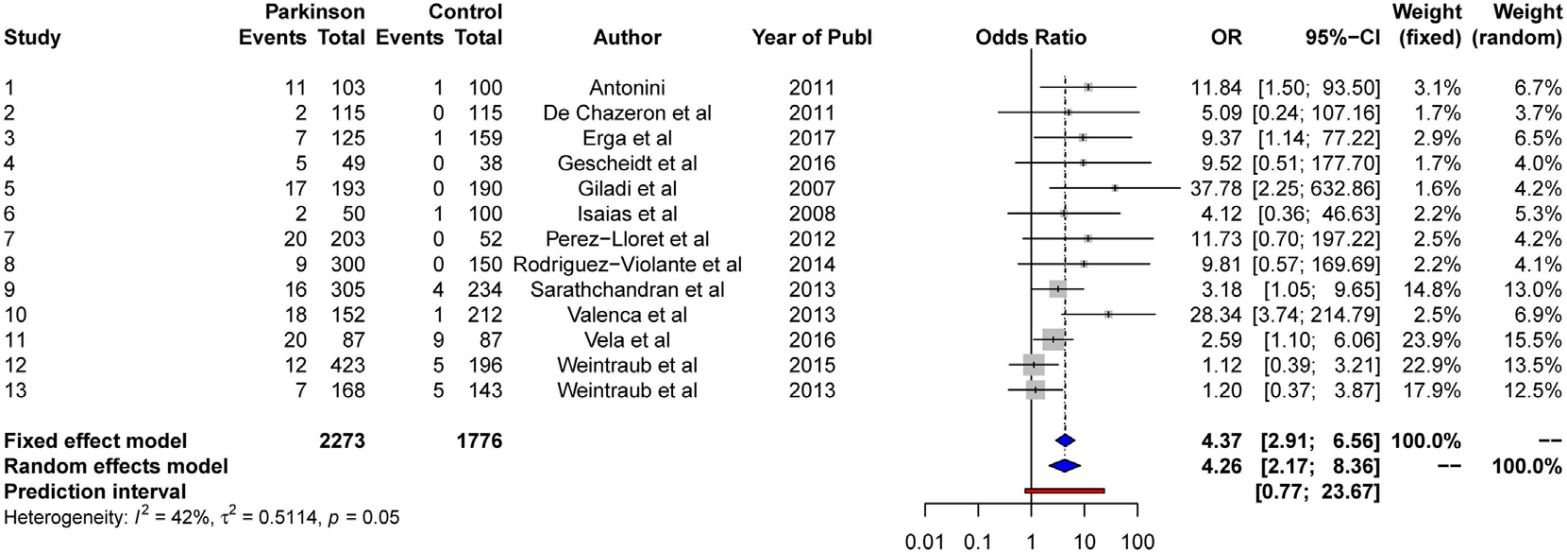
Forest plot for hypersexuality.

The results from the random-effects model on *hypersexuality* showed a significant *OR* estimate of point 4.26, 95% CI [2.17, 8.36]. The *Q*-statistics were non-significant (*Q* = 20.8, *p* > 0.05), indicating non-significant heterogeneity between the studies. The between-study variance, Tau2, was 0.51, and the percentage of unexplained between-study variance *I*^2^ = 42.4%, 95% CI [0.00, 70.0]. This indicated moderate unexplained between-study variance with respect to hypersexuality. See Figure 6 for a forest plot of the results.

**Figure 7:**
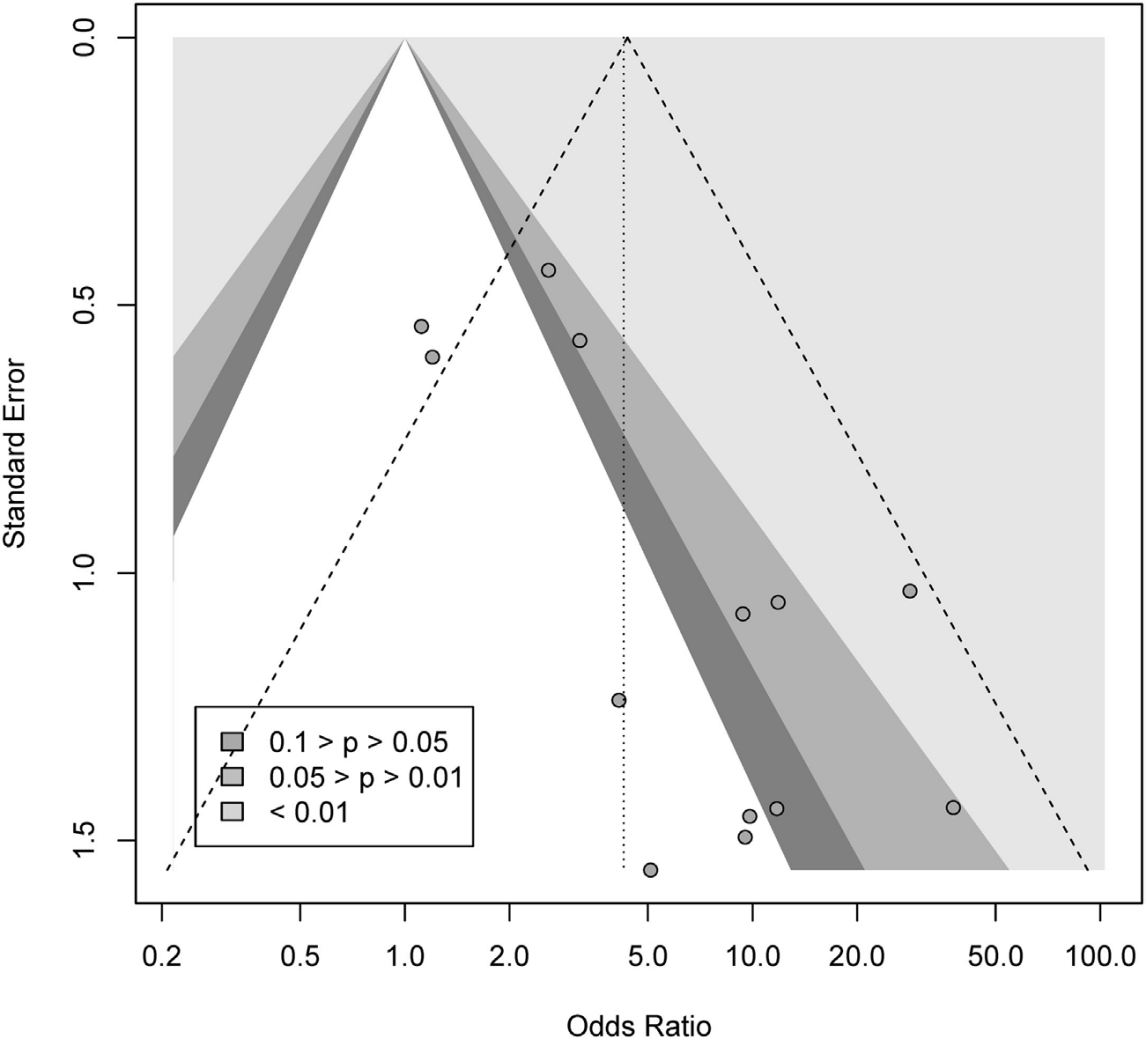
Contour-enhanced funnel plot for hypersexuality.

We conducted a contour-enhanced funnel plot estimating publication bias. As seen from Figure 7, there seem to be several studies with large standard error lacking in the lower white area, as in comparison to studies with low standard errors lacking in the gray area, in order to create more balance in the figure. Thus, this may indicate both a small-study effect and a publication bias. However, the Rüker’s test was non-significant (*t* = 1.4, *p* > 0.05), indicating no publication bias.

The Rosenthal’s fail-safe *N* test was significant (*p* < 0.0001), indicating that 165 non-significant studies would be needed for the random-effects model to be non-significant. The univariate testing of possible moderators of heterogeneity resulted in two significant bivariate models: disease duration and medically treated PDs.

#### Mixed-Effects Model

The results from the meta-regression analysis showed a significant log-*OR* point estimate of β0 = 0.81 (*OR* = 2.48, 95% CI [1.02, 5.00]). The overall moderator model was significant, QM = 7.36 (*p* = 0.011). Both of the moderators included were significant. Disease duration was significant at the 0.05 level, *OR* = 1.20, 95% CI [1.02, 1.40]. Treated PDs was also significant at the 0.05 level, *OR* = 2.63, 95% CI [1.04, 6.61]. The test for residual heterogeneity was non-significant, QE = 7.60, *p* = 0.68.

The model accounted for 96.5% (*R^2^*) of the heterogeneity, and the percentage of residual heterogeneity, *I*^2^, was 2.39%. Tau2, or residual heterogeneity, was 0.02 (SE = 0.28). The QQ-normal plot indicated a normal distribution of the residuals.

### Shopping

#### Random-Effects Model

**Figure 8:**
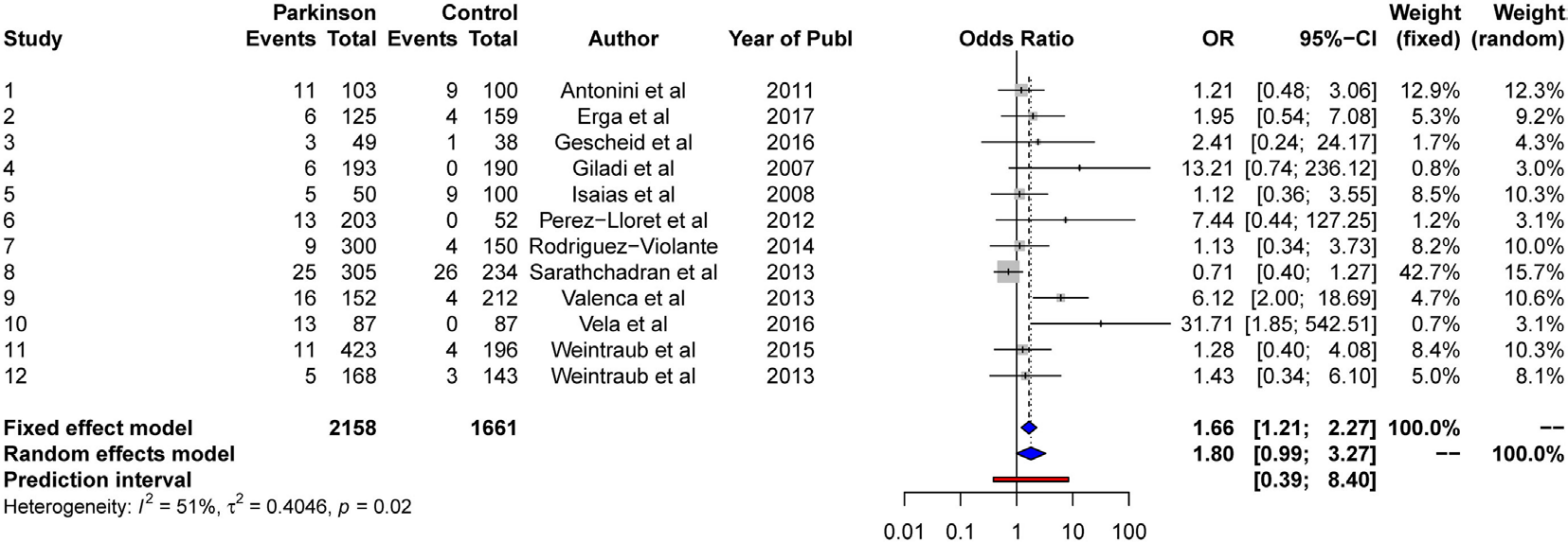
Forest plot for shopping.

The results from the random-effects model on *shopping* showed a non-significant *OR* estimate of point 1.80, 95% CI [0.99, 3.27]. The *Q*-statistics were non-significant (*Q* = 22.3, *p* > 0.05), indicating no significant heterogeneity between the studies. The between-study variance, Tau2, was 0.40, and the percentage of unexplained between-study variance *I*^2^ = 50.8, 95% CI [4.7, 74.6]. This indicates a moderate level of between-study variance with respect to shopping. See Figure 8 for a forest plot of the results. Due to the non-significant intercept, no further analyses were conducted for shopping.

### Eating

#### Random-Effects Model

**Figure 9:**
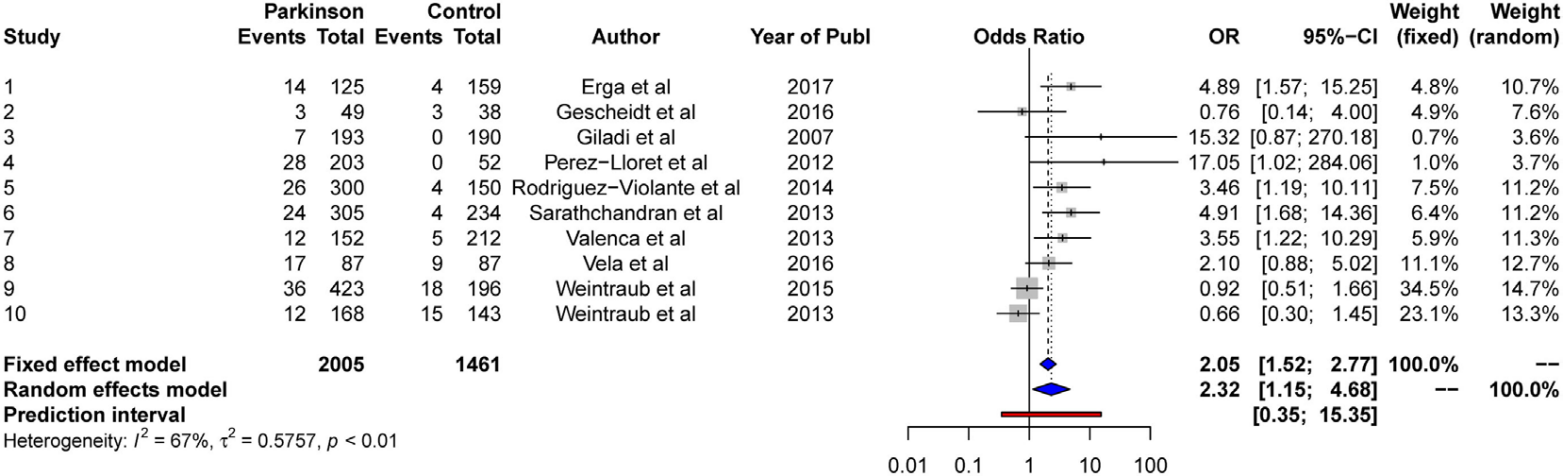
Forest plot for eating.

The results from the random-effects model on *eating* showed a significant *OR* estimate of 2.32, 95% CI [1.15, 4.68]. The *Q*-statistics were significant (*Q* = 27.1, *p* < 0.001), indicating significant heterogeneity between the studies. The between-study variance, Tau2, was 0.58, and the percentage of unexplained between-study variance *I^2^* = 66.8, 95% CI [35.2, 82.9]. This indicates high unexplained between-study variance with respect to eating. See Figure 9 for a forest plot of the results.

**Figure 10:**
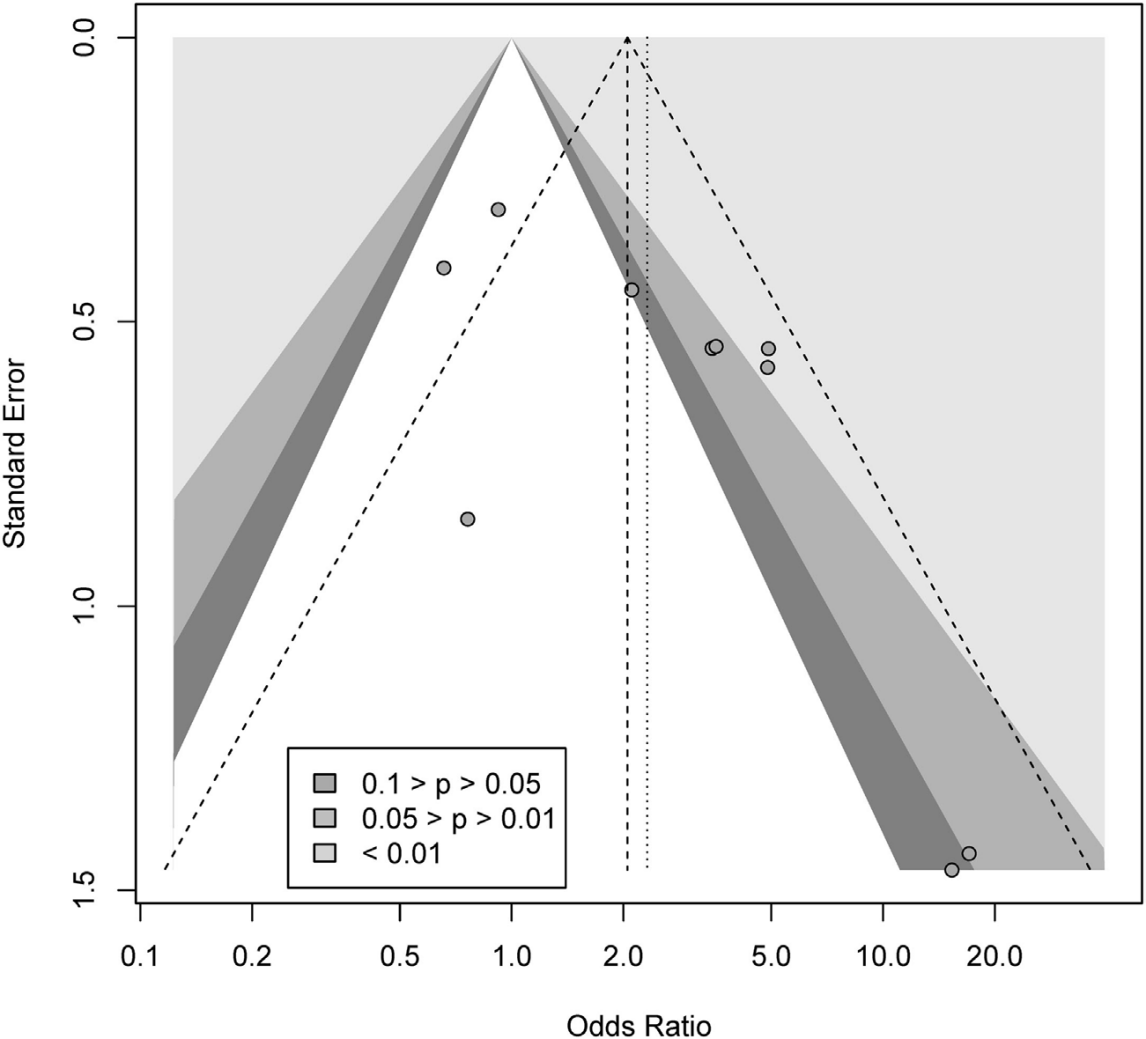
Contour-enhanced funnel plot for eating.

We conducted a contour-enhanced funnel plot estimating publication bias. As seen from Figure 10, there seems to be about an equal number of studies lacking in the upper white area, as compared with studies with low standard error lacking in the upper gray area, in order to create more balance in the figure. Thus, this may indicate a small-study effect, but perhaps not due to publication bias. Also, the Rüker’s test was non-significant (*t* = 0.14, *p* > 0.05), indicating no publication bias.

The Rosenthal’s fail-safe *N* test was significant (*p* < 0.0001), indicating that 64 non-significant studies would be needed in order for the random-effects model to be non-significant.

The univariate testing of possible moderators of heterogeneity resulted in three significant univariate models: number of patients using DAs, number of patients using l-dopa and being medically treated for PD. The correlation between the two former moderators was 0.91, thus we only included being medically treated for PD in the mixed model.

#### Mixed-Effects Model

The results from the meta-regression analysis showed a non-significant log-*OR* point estimate of β0 = −0.20 (*OR* = 0.82, 95% CI [0.47, 1.42]). The intercept β0 refers in this model to *de novo* patients or non-medically treated patients. The results indicate that *de novo* PD patients do not differ in comparison with normal controls with respect to eating problems. The overall moderator model was significant, QM = 18.5 (*p* = 0.002), and as stated above, being medically treated for PD (*OR* = 4.06, 95% CI [1.92, 8.58]) was significant. The test for residual heterogeneity was non-significant, QE = 7.89, *p* = 0.44. The model accounted for 100% (*R^2^*) of the heterogeneity, and the percentage of residual heterogeneity, *I*^2^, was 0.0%. Tau2, or residual heterogeneity, was 0.00 (*SE* = 0.13). The QQ-normal plot indicated a normal distribution of the residuals.

### Punding

#### Random-Effects Model

**Figure 11:**
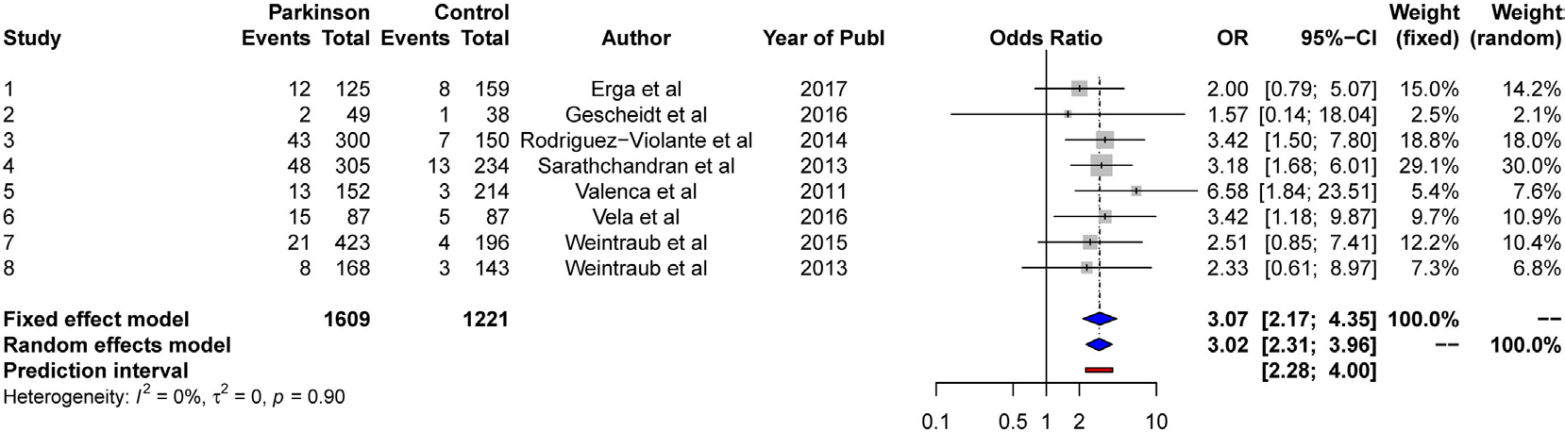
Forest plot for punding.

The results from the random-effects model on *punding* showed a significant *OR* of point 3.02, 95% CI [2.31, 3.96]. The *Q*-statistics was non-significant (*Q* = 2.88, *p* > 0.05), indicating no significant heterogeneity between the studies. The between-study variance, Tau2, was 0.0, and the percentage of unexplained between-study variance was: *I*^2^ = 0.0%, 95% CI [0.00, 21.3]. See Figure 11 for a forest plot of the results.

**Figure 12:**
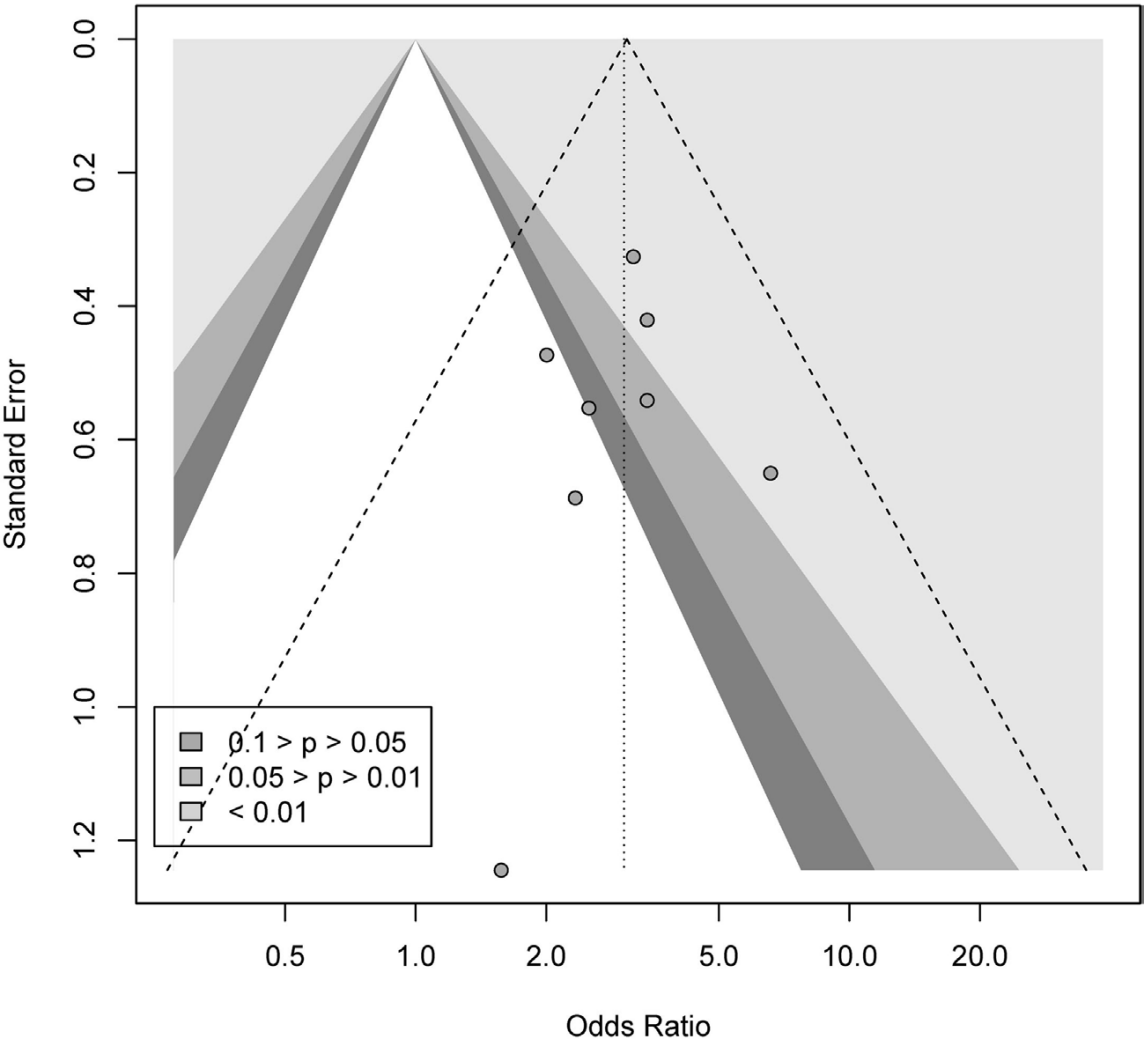
Contour-enhanced funnel plot for punding.

We conducted a contour-enhanced funnel plot estimating publication bias. As seen from Figure 12, the plot indicated a larger number of studies with standard error lacking in the gray area, as in comparison to studies with standard errors lacking in the white area, in order to create more balance in the figure. Thus, this does not indicate a small-study effect or publication bias. Due to the low number of studies (<10), no Rüker’s test was conducted.

Due to the *I*^2^ estimate, no mixed-effects model was conducted. Hence, no further testing was applied for punding.

### Hobbying

#### Random-Effects Model

**Figure 13:**
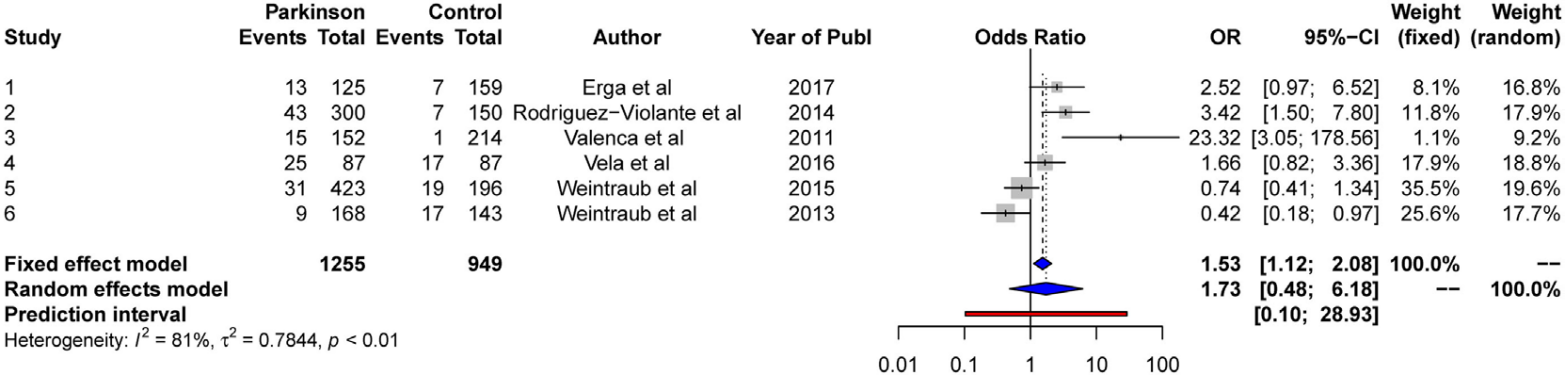
Forest plot for hobbying.

The results from the random-effects model on *hobbying* showed a non-significant *OR* at point 1.73, 95% CI [0.48, 6.18]. The *Q*-statistics were significant (*Q* = 26.5, *p* < 0.0001), indicating significant heterogeneity between the studies. The between-study variance, Tau2, was 0.78, and the percentage of unexplained between-study variance *I*^2^ = 81.1, 95% CI [59.5, 91.2]. This indicates large unexplained between-study variance with respect to hobbying. See Figure 13 for a forest plot of the results.

As two of the six *hobbying* studies included are using *de novo* PDs, we decided to use patients being medically treated for PD as a moderator.

#### Mixed-Effects Model

The results from the meta-regression analysis showed a non-significant log-*OR* point estimate of β0 = −0.53 (*OR* = 0.59, 95% CI [0.20, 1.79]). The intercept β0 refers in this model to *de novo* patients, or non-medically treated patients. The results indicate that *de novo* PD patients do not differ in comparison with normal controls with respect to hobbying.

The overall moderator model was significant, QM = 8.53 (*p* = 0.04), and being medically treated for PD (*OR* = 4.66, 95% CI [1.08, 20.0]) was significant. The test for residual heterogeneity was non-significant, QE = 7.59, *p* = 0.11. The model accounted for 92.6% (*R^2^*) of the heterogeneity, and the percentage of residual heterogeneity, *I*^2^, was 27.60%. Tau2, or residual heterogeneity, was 0.00 (SE = 0.28).

The QQ-normal plot indicated a normal distribution of the residuals.

## Discussion

This is, as far as we know, the first meta-analysis to examine ICDs in PD using case–control studies. With this quantitative synthesis we wanted to summarize the existing research and extend earlier reviews in order to better understand, and quantify the association between ICDs in PD. The estimated *OR*s ranged between 2.07 for having any ICD, and 4.26 for hypersexuality. These results demonstrate that ICDs are significantly associated with PD, which is in line with previous narrative reviews (17, 23, 26).

In several of the random-effects models, there was significant heterogeneity, with high between-study variations, as shown with index *I*^2^. This implies that there are important between-study characteristics that moderate the between-study estimates of the true effect. Using meta-regression models, we identified sources of between-study variations by modeling moderators of heterogeneity. This is vital for the development of new hypotheses because moderators can identify factors that may be of significance and thus, effectively target treatment and prevention strategies (66).

The results suggest that impulsive behavior is not an invariant feature of PD, but varies by a number of important explanatory variables including; being medically treated for PD and disease duration. Still, it should be noted that the associations are not at the individual level, but at the study level, or as a moderation of between-study estimates. This distinction is important. That said, DA treatment has been suggested as a risk factor for ICDs (67), and our results seem to support this notion. Number of patients using Levodopa, however, was only significant in a bivariate model for eating. Still, previous studies have identified levodopa as a possible risk factor for ICDs (17, 23). However, and notably, the moderator: being medically treated for PD covers all types of medication, l-dopa included. Thus, the likely explanation for the lack of significance is the lack of power, or the relatively low number of studies included in the present meta-analysis. Thus, for *shopping* the random-effects estimate was almost significant, with the 95% CI ranging from 0.99 to 3.27. Notably, the fixed-effects model for *shopping* was significant (*OR* = 1.66, 95% CI [1.2147, 2.2742]), meaning there is an effect for the studies included, but that the effect cannot be generalized to the wider population of studies. However, overall, it should also be noted that our mixed-effects models explained a significant amount of heterogeneity in the different models, to the point where there was no heterogeneity left to explain.

Still, and as mentioned in Section “Introduction,” ICDs in PD do have a multifactorial nature to be considered. Several variables regarded as risk factors were not included in our data, e.g. novelty-seeking personality traits, personal or family history of addictions prior to PD diagnosis, comorbid psychiatric disorders, and cognitive dysfunctions (e.g., decision making, set-shifting, etc.) (68). It is important to stress the multifactorial nature of ICDs in PDs, as not all patients developing ICDs are exposed to dopaminergic medications (69). Thus, other explanations and mechanisms should be identified, especially related to individual genetically vulnerability. Genetically, studies have shown that dopaminergic, opioid, and serotonergic genotypes are related to ICDs in PDs. In addition, environmental and cultural factors may also contribute (70).

### Publication Bias

As the presented results show, two of the models (hypersexuality and having any ICD) had some possibility of publication bias according to the contour-enhanced plots. For the other models, the plots indicated very little publication bias. Notably also, the Rüker’s test was not significant for any model, supporting an interpretation of lack of publication bias within the models/results. Regarding the Rosenthal’s fail-safe *N* from 64 (*eating*) to 165 (*hypersexuality*) to 226 (any ICD) non-significant studies would needed to be included to make the overall results non-significant. Overall, this suggest stability of results.

### Strengths and Limitations

The inclusion of observational studies in meta-analyses has led to questions about validity of results. Observational studies are in general criticized as susceptible to subjective interpretations, unidentified confounding variables, and risk modification. The analysis of the data from the meta-analysis itself is accordingly also vulnerable to subjective bias (66, 71).

We made use of random-effects models, which are generally more vulnerable to publication bias than fixed-effects estimates. On the other side, using a fixed-effects model would assume only within-study errors, or no between-study variation, which is an assumption that is seldom met (55).

We did not conduct a subgroup sensitivity analysis due to the low number of studies included. However, with a larger sample of studies, this would enable researchers to evaluate the impact of heterogeneity versus the impact of publication bias in the estimates (72).

When evaluating the methods used, we depended on data reported in the included studies. We did not contact the authors if methods were poorly reported. Unclear reporting does not necessarily mean insufficient study administration, but can limit the understanding of the study (17).

Although it can be challenging, one of the intents of a meta-analysis is to find and assess all studies meeting a set of inclusion criteria. Many studies may not have been published and can systematically differ from the published ones. Significant, positive findings are shown to be more likely to be published compared to small, negative studies, hence this may lead to publication bias (66, 72, 73). Still, attempts to retrospectively gather information from unpublished studies does not seem to address publication bias sufficiently (74).

A potential bias can arise due to excluding studies reported in languages other than English. However, language bias is reported to only have modest or no effect (75).

It should also be noted that direct comparisons of ICD prevalence in various studies are complicated by different assessment methods, with emphasis put on different time frames (76).

### Implications

The results of the present meta-analysis show that there is a significant chance of developing ICDs in PD patients compared to healthy controls. The investigation of possible moderators of heterogeneity resulted in two variables (being medically treated for PD and disease duration) which are significant in mixed models. This is supported by previous studies looking into risk factors for development of ICDs in PD (13, 26, 28). The present results can as such have implications for how PD patients should be met and treated. Thus, practitioners should routinely ask about behavioral changes during assessment, and relevant countermeasures such as down-titration of DA and cognitive behavioral therapy should be considered. Warning patients about behavioral side-effects of DA should be implemented, as least for vulnerable PD patients (77–79).

## Conclusion

The present results show a significant relationship between ICDs and PD. Duration of PD and being medically treated for PD are moderators positively associated with ICDs in PD patients. Proper assessment during physician consultations is critical as ICDs can significantly harm the overall social functioning and personal relationships of this patient group. The use of DAs seems to pose an especially high-risk factor for ICD development, thus pharmacological treatment needs to be carefully monitored. Caretakers and relatives should be involved, as patients may lack insight, or find their behavior embarrassing.

Finally, conducting a precise meta-analysis is dependent on the quality of the included research articles. As there always will be a risk of publication bias, results must be interpreted with caution.

## Author Notes

Ref. (15, 16, 33, 42, 44–55) are studies included in the meta-analysis.

## Author Contributions

HM, YM, and SK did the literature research and study coding. HM did the statistical analysis. All authors have significantly contributed ideas, commenting on the manuscript and reviewing the paper during the writing process.

## Conflict of Interest Statement

The authors declare that the research was conducted in the absence of any commercial or financial relationships that could be construed as a potential conflict of interest.
